# Are the Quality of Organizational Changes Associated with Expected Retirement-Age Among Senior Employees?

**DOI:** 10.1007/s10926-024-10241-8

**Published:** 2024-10-24

**Authors:** Karen Albertsen, Annette Meng, Emil Sundstrup, Peter Nielsen, Flemming Pedersen, Lars Louis Andersen

**Affiliations:** 1 Team Working Life, Høffdingsvej 22, 1. Sal, 2500 Valby, Denmark; 2 National Research Centre for the Working Environment, 2100 Copenhagen, Denmark; 3 Department of Politics and Society, Aalborg University, 9220 Aalborg, Denmark

**Keywords:** Early retirement, Retirement intentions, Reorganizations, Restructuring, Downsizing, Implementation

## Abstract

**Objective:**

Major organizational changes may be associated with both positive and negative uncertainty in working life. This study described the prevalence of organizational changes (reorganizations or round of layoffs) within different job functions in Denmark and investigated whether quality of the implementation process (measured as “information”, “involvement” and “consent”) was associated with employees’ expectations regarding retirement age.

**Methods:**

A representative sample of older Danish employees ≥ 50 years (*n* = 12,269) replied to a questionnaire survey in 2020. In cross-sectional analyses, we compared employee’s expected retirement age being either not exposed to organizational changes or exposed to implementation processes of high, moderate or low-quality, respectively. Analyses were further stratified for job function: office work, work with people and work in the field of production.

**Results:**

More than half (56%) of the employees had experienced organizational changes within the past 2 years, and 23% of those effected reported that the changes had led to considerations of earlier retirement. Organizational changes were most prevalent within office work, and least prevalent within the job function working with people. The analyses showed significantly lower expected retirement age when the implementation process had been of moderate (mean reduction of 0.45 years) or low quality (mean reduction of 0.71 years) compared to high quality implemented changes.

**Conclusions:**

Experiences of organizational change processes of moderate or poor quality were associated with expectations of earlier retirement, while well implemented changes were not. This study underscores the importance of good implementation when changes at the organizational level are needed.

## Introduction

The workforce is aging and therefore the need to create workplaces that support long, and healthy working lives increases. A range of different job-related factors may influence the individual-level decisions of retirement by acting as barriers (e.g., high physical and cognitive work demands, poor possibilities for development, low appreciation at work, low affective commitment) or motivators (e.g., influence, lower workplace, more time to complete tasks) for prolonged working life after the pensionable age [1–6].

Cultural organizational factors at the workplace level may also contribute to the intentions of retirement. Among other, organizational pressure for early retirement as well as the social timing of retirement play important roles for the decision of retirement [7]. In a scoping review, [3] however, conclude that most investigations are conducted on individual-level predictors, while research on organizational-level predictors is more scattered.

Organizational changes, reorganization as well as downsizing as strategical managerial tool to obtain improved efficiency and organizational performance has become common at the workplaces of today [8]. While this may lead to immediate innovation or economic savings in terms of salaries, such strategies can also backfire in various ways. First, downsizing may lead to loss of important knowledge due to dismissal of employees. Particularly, some older and experienced employees may either choose to or are forced to retire because of—or to prevent—dismissal. Second, the implementation process of the organizational change may be associated with negative as well as positive effects for the remaining employees. Thirdly, the outcome of the reorganization may lead to negative as well as positive changes for the employees, supporting decisions of either early or postponed retirement.

Studies have found negative effects for the remaining employees after organizational changes, such as mental distress [9], particularly when employees have been exposed to repeated organizational change [9, 10] p. 12). Other studies have found organizational change related to increased work intensity and physical strain [11].

However, only few studies have focused directly on the association between company reorganization or downsizing and early retirement. A Dutch study did not find restructuring prospectively associated with early retirement [4], while a study from Denmark found exposure to organizational change (change of management, merging, demerging, and relocation) associated with an increased rate of non-disability early retirement [12]. The same group of researchers found that organizational change at the level of work-unit prospectively predicted lower social capital [13], and further that lower levels of work-unit social capital, organizational justice, and quality of management, was associated with increased rates of early retirement [12]. Thus, workplace social capital may play an important role in the process were organizational change influence retirement decisions.

The way organizational changes are implemented may ameliorate the side-effects. Studies have proposed the concept of ‘responsible downsizing’ to describe the actions, practices and strategies adopted by organizations to ameliorate the negative effects of redundancy [14]. Among others, studies have emphasized the importance of truthfulness and transparency throughout the decision-making processes [15], as well as the importance of involvement of workers’ representatives, the way the workforce reduction was communicated and how the future of the workplace was framed [16]. Thus, the quality of the implementation process may be crucial for the impact of the organizational change on the work environment and wellbeing of the employees.

Together, the previous studies have indicated, that major organizational changes causing negative effects on the work environment, are likely to push and enforce intentions of an earlier retirement among older employees. However, many of the previous studies are conducted on a limited number of participants (i.e., small-scale) and not representative of the general working population of older workers.

The objectives of the present study, representative of + 50-year employees in Denmark, were 1. To describe the prevalence of organizational changes within three distinct job functions (office work, work with people, and work in the field of production) 2. to investigate whether the assessed quality of the implementation of organizational changes was associated with older employees’ expectations regarding retirement age, and 3. whether the association differed according to the employees’ job functions.

This study hypothesized (1) That experiences of reorganization and round of layoffs would be associated with earlier expected retirement age among senior employees, and (2) that the quality of implementation would modify the association with earlier expected retirement age.

## Methods

The study is based on the employee survey in the SeniorWorkingLife study, 2020 [17]. A representative sample of 18,000 employed Danes ≥ 50 years were invited to participate in the part of the survey that concerned current working situation. For the analyses in this article, we included only currently employed wage earners. Employed wage earners were defined based three previously described criteria (ibid.).

### Subgroups of the Study Population

The following question was used to determine the job function: “What do you work with first and foremost in your daily work?” with the following three response categories: (1) office work, administration, analysis, IT; (2) work with people, service, care and (3) work with processing, producing, or moving things. For further analysis, the respondents were stratified in relation to these response categories: response category 1 was designated as office work; response category 2 was designated as work with people; response category 3 was designated as work in the field of production (ibid.). In total, 12,269 participants responded to the questions about organizational change and were included in the analysis (5718 with office work, 3222 working with people, 1558 working in the field of production).

### Predictors

Exposure to *organizational change* was measured by two questions: “Has your workplace been reorganized within the last 2 years (e.g., the workplace has been merged with another workplace, or departments have been merged)?” and “Has a round of layoff been conducted at your workplace in recent years?”. Both questions had the response categories: (i) No; (ii) Yes, 1 time and (iii) Yes, several times. Responding ‘yes’ to at least one of these was defined as ‘an organizational change’ and led to further questions.

A further question measured whether the experienced changes had led to considerations about retirement: “Do the changes at your workplace have an impact on your considerations regarding retirement?” with the response options: “Yes, I am thinking of retiring later”, “Yes, I am thinking of retiring earlier” and “no”.

For those experiencing at least one organizational change, the *quality of the implementation* of organizational changes was measured by a scale covering 4 items:

“Has the management informed employees adequately about the changes in the workplace?”, “Have the employees been sufficiently involved in connection with the changes?”,” Are you generally satisfied with the way management has handled the changes?” and “Do you understand the management's reasons for implementing the changes?”. All items had a 5-point response scale ranging from 1 = to a very high degree to 5 = not at all. Subsequently, responses were recoded to 0–100 (0 ‘not at all’, 100 ‘to a high degree’) and averaged for the four questions. Cronbach’s alpha for the scale was 0.87. The average score defined the ‘subjective quality of the implementation of organizational changes’.

Based on responses to the questions on organizational changes and the scale on quality of implementation, an index was constructed covering four categories: 0) No reorganizations in recent years (replying ‘no’ to the two first questions about reorganization and firing); (1) Reorganizations, handled well by management (replying ‘yes’ to at least one of the first two questions and having a ‘quality of implementation’ score of 60–100); (2) Reorganizations, handled moderately well by management (same as 1, but scoring 40–59 on ‘quality’ and (3) Reorganizations, handled poorly by management (same as 1, but scoring 0–39 on ‘quality’).

### Outcome

The outcome variable of expected retirement age was assessed by the open-ended question: “At what age do you expect to leave the labor market completely?”. Age was measured as a continuous variable.

### Covariates

The statistical model was adjusted for the following potential confounders: age (years), gender (male/female), education (the highest attained educational level and drawn from a national register handled by Statistics Denmark: (1) primary school or high school; (2) vocational training (3) higher education); body mass index (BMI) (1) BMI 18-25(e), (2) BMI 25-30(e), (3) BMI 30-35(e), (4) BMI 35-40(e), (5)BMI >  = 40, (6) BMI < 18); smoking status (No/Yes); alcohol intake (drinking more or less than the recommended thresholds based on the National Board of Health recommendations); family type: (1) singles without children, (2) singles with children, (3) couples without children and (4) couples with children.

### Statistics

We used the General Linear Model (GLM) procedure of SAS 9.4 (SAS Institute, Cary, NC, USA) to examine the association between the quality of implementation of organizational changes and expected retirement age. The dependent variable was the expected retirement age. The main predictor variable was a categorical variable representing the quality of implementation of organizational changes. The statistical model was adjusted for the potential confounders described above: age, gender, educational level, body mass index, smoking status, alcohol intake, and family type. The analysis was weighted using model-assisted weights to ensure the representativeness of the sample. Least-squares means were calculated for the expected retirement age for each level of the quality of implementation variable. The main analysis was further stratified for job function (office, production, people).

## Results

The prevalence of experienced round of layoffs and reorganizations within the three job-function categories are presented in Table 1. The results show that experiences of one or more rounds of layoffs within the last 2 years were more prevalent among participants with office work (44%) and in the field of production (45%) than it was among those working with people (27%). Experiences of one or more reorganization were more prevalent among office workers (50%) than among the job functions working with production (32%) and people (35%).

**Table 1:** Characteristics of the entire study sample (*N* = 12.855) and stratified by job function category (office work, work with people, work in the field of production)

	Total		Office		People		Production	
	*N*	Percent or Mean (SE)	*N*	Percent or Mean (SE)	*N*	Percent or Mean (SE)	*N*	Percent or Mean (SE)
Age (years)	12,855	57.0 (0.07)	5917	56.7 (0.11)	3385	57.2 (0.13)	1660	56.9 (0.20)
Gender								
Female	5580	46	2710	47	2260	70	208	13
Male	6762	54	3068	53	952	30	1378	87
Education								
Primary school or high school	1961	15	453	8	451	13	534	32
Vocational training	6812	53	3350	57	1358	40	1071	64
Higher education	4082	32	2114	36	1576	47	56	3
Smoking								
No	10,235	82	5017	86	2612	79	1226	77
Yes	1979	18	682	14	597	20	326	23
Alcohol intake								
At or below National threshold	9959	87	4591	85	2614	87	1313	91
Above National threshold	1818	13	995	15	444	13	177	9
BMI (kg/m2)	12,088	26.7 (0.08)	5634	26.5 (0.12)	3168	26.3 (0.15)	1542	27.7 (0.21)
Family type								
Singles without children	2454	21	993	18	747	22	346	25
Singles with children	425	4	216	5	115	5	42	3
Couples without children	7107	49	3205	48	1885	51	938	49
Couples with children	2869	26	1503	29	638	22	334	24
Experienced changes	5860	56	3532	62	1492	46	836	54
Experienced > 1 rounds of layoffs	1828	17	1157	20	313	10	358	23
Experienced 1 round of layoffs	2302	22	1403	24	555	17	344	22
No round of layoff	6375	61	3167	55	2352	73	858	55
Experienced > 1 reorganization	1837	17	1325	23	334	10	178	11
Experienced 1 reorganization	2665	25	1524	27	813	25	328	21
No reorganization	5996	57	2869	50	2075	64	1052	68
Quality of implementation								
No organizational changes	5604	45	2179	37	1.720	51	718	43
Changes with high quality implementation	2058	17	1240	22	419	14	186	12
Changes with moderately quality implementation	2484	21	1323	24	569	19	324	21
Changes with low quality implementation	2106	18	976	16	508	16	329	24
Changes and considerations of retirement								
Experience of changes have led to considerations about earlier retirement	1348	23	749	21	390	26	209	25
Experience of changes have led to considerations about later retirement	153	3	53	1	52	3	48	6
Experience of changes have not led to considerations about retirement	4357	74	2730	77	1050	70	577	69

For 21–25% of those who had experienced organizational changes at the worksite, the changes had led to considerations of earlier retirement. For a small percentage of employees (1–6%) the changes had led to considerations of later retirement. No significant differences in the proportion of employees reporting considerations of earlier or later retirement were observed between the three job-function categories.

Table 2 illustrates the association between quality of implementation of organizational change and expected retirement age for the entire study sample. The analysis shows, that those reporting changes with poor quality implementation had lower expected retirement age (age of 67.37) than those reporting high quality implementation (age of 68.08) or no changes (age of 67.96), respectively.

**Table 2:** Mean expected retirement age and 95% CI for each of the four groups of quality of implementation and for participants working with office work, production, or people, respectively

Quality of change	Total expected retirement age and (95% CI)	Office expected retirement age and (95% CI)	People expected retirement age and (95% CI)	Production expected retirement age and 95% CI
1.No changes	67.96 (67.74–68.17)	68.46 (68.13–68.79)	66.66 (66.32–66.99)	67.93 (66.95–68.91)
2.Changes with high quality implementation	68.08 (67.83–68.33)	68.28 (67.91–68.64)	67.18 (66.78–67.58)	67.72 (66.60–68.83)
3.Changes with moderate quality implementation	67.63 (67.39–67.87)	67.90 (67.55–68.26)	66.70 (66.33–67.07)	67.15 (66.13–68.16)
4.Changes with poor quality implementation	67.37 (67.12–67.61)	67.64 (67.26–68.02)	66.60 (66.21- 66.98)	67.04 (66.04–68.05)

As shown in Table 3, employees exposed to poorly implemented changes, reported on average approximately 0.7 years lower expected retirement age than those exposed to well implemented changes. Employees exposed to moderately well implemented changes reported on average 0.5 years lower expected retirement age than those exposed to well implemented changes. These differences were statistically significant.

**Table 3:** Differences between means of expected retirement age for the groups experiencing no changes, high, moderate or low quality changes, 95% CI for each of the differences and *p*-values

	Difference between means	95% CI	*p*-value
No (1) vs high (2)	− 0.13	− 0.31–0.06	0.2
No (1) vs moderate (3)	0.36	0.16–0.50	0.0002
No (1) vs low	0.59	0.40–0.77	< .0001
High (2) vs moderate (3)	0.45	0.24–0.66	< .0001
High vs (2) vs low (4)	0.71	0.49–0.93	< .0001
Moderate (3) vs low (4)	0.26	0.06–0.47	0.01

Post hoc analyses segregated on the three job-function categories showed similar trends in all groups (see Table 2). Expected retirement age were between 0.4 and 0.6 years lower for employees exposed to moderately well implemented changes compared to well implemented changes and between 0.6 and 0.7 years lower for employees exposed to poorly implemented changes compared to well implemented changes. The differences within each of the job-function categories were not statistically significant.

However, for employees with office work and in the field of production, the largest differences were found between the group with no changes and the group with poorly implemented changes. In the group with office work this difference in expected retirement age was statistically significant (0.8 years). In the group working in the field of production the difference was of same size but not statistically significant (0.9 years). Among employees working with people, the largest difference was found between the group with well implemented changes compared to the group with poorly implemented changes (0.6 years and NS).

## Discussion

### Main Findings

More than half of the employees had experienced organizational changes within the past 2 years, and for many participants, this had led to considerations of earlier retirement. The analyses showed a lower expected retirement age when the changes had been moderately or poorly implemented compared to well implemented changes or no changes. Thus, the results support our hypotheses: (1) that experiences of reorganization and round of layoffs were associated with earlier expected retirement age among senior employees, and (2) that the quality of implementation modified the association with earlier expected retirement age.

### Reorganizations and Retirement Expectations

The results showed that a considerable part of the employees had experienced at least one reorganization or round of layoff within the past 2 years. The prevalence for reorganizations were lowest in the group of job functions working with people, and highest in the group with office work. For rounds of layoffs, it was equally high among employees with office work and in the field of production. Across all job-function categories, the changes had for about a quarter of the employees led to considerations of earlier retirement, and only for a small part had led to considerations of later retirement.

To our knowledge there are only few studies on this topic, and they show somewhat conflicting results. One study found change of management and merging, or relocation of work units prospectively associated with a higher likelihood of early retirement (before the official retirement age of 65 years) [12]. Another study [4] did not confirm a prospective association between restructuring and early retirement.

Results from our study suggest that experiences with reorganization are very common at the Danish labor market. More than half in this large representative sample of older employees had been exposed to reorganizations or rounds of layoffs within a two-year period. And for approximately a quarter of the exposed, this had led to considerations of earlier retirement. The considerations may not necessarily lead to actual retirement (section on strength and limitations), but given that the exposure is so common, it is nevertheless likely that reorganizations and rounds of layoffs on average contribute to earlier retirement.

### Quality of Implementation and Retirement Expectations

The analyses showed statistically significant lower expected retirement age for older employees exposed to moderately and poorly implemented organizational changes compared to well implemented changes or no changes. Although the numerical differences on average were of moderate size (0.3–0.7 years), the total expected ‘loss’ of work-productive years at the societal level may be considerable.

Breinegaard et al. [12] found that experiences of change of management and merging or relocation of work units increased the rate of early retirement among health-care workers. However, after adjusting for social capital, organizational justice and quality of management, the rate of early retirement was still significantly higher among employees who experienced change of management, but the remaining types of organizational change had no significant effect. These results may suggest an interplay between the social capital and the effect of organizational changes on retirement. In our study we found that poorly implemented organizational changes were associated with lower expected retirement age. Other studies have found social capital to be associated with among others work engagement [18] and job-satisfaction [19]. Thus, it may be hypothesized, that organizations with low quality implementation of change process at the same time are organizations with lower social capital, organizational justice, and quality of management, and accordingly that a negative spiral may occur in which workplaces with low social capital may implement changes in ways that reduce engagement and loyalty and increase retirement intentions among older workers, and in turn reduce the social capital.

Previous studies have highlighted characteristics of good management practice in relation to organizational change processes. Such factors include a shared change vision and strategy, effective and constant communication, engagement and commitment of stakeholders [20] truthfulness, uprightness and transparency throughout the decision-making process [15], offering a choice to leave voluntarily [16], establishing trust and rewarding and recognizing surviving employees [21]. These guidelines comply with the ways we have measured the quality of implementation in this study and may suggest that a profound planning thorough the implementation process can make a huge difference for the potential negative effect of reorganizations and downsizing on the employees. However, in practice this may be difficult.

All in all, our study adds to the previous studies by suggesting an association between the experienced quality of organizational changes and the expectations to retirement age.

### Differences Between Job-Function Categories

Our results suggested a higher prevalence of rounds of layoffs for employees with office work and work in the field of production compared to employees working with people, and a higher prevalence of reorganizations within work with office work than in the two other job-function categories.

Further, results suggested slightly different patterns for the three job-function categories, with the largest difference in expected retirement age due to quality of implementation of changes for the category ‘office work’ and ‘work within production’ and with the smallest difference for those ‘working with people’. Among those ‘working with people’, the expected retirement age was higher for those exposed to well implemented changes than it was among those with no changes. In the other job-function categories, no changes were associated with the highest expected retirement age.

There may be various explanations for these differences between job-function categories. It could be hypothesized that employees with office work on average have more opportunities to choose by themselves when they want to stop working [1], and therefore are more likely to retire early if they experience too many, and particularly to poorly implemented reorganizations. The job functions with office work and with production are both highly exposed to rounds of layoffs making it more likely either that employees expect that they will be fired themselves, and accordingly expect earlier retirement, or that employees are more likely to choose for themselves to leave early to avoid being fired. The results indicate that employees working with people are less exposed to changes (both reorganizations and rounds of layoffs) and may accordingly be more likely to welcome the well implemented organizational changes, when they come, and less likely to make the organizational changes influence retirement age.

Because work with people in a Danish context are often under the public sector, the results may reflect findings from a Swedish study suggesting that “*Not all organizational change resulted in a poorer work environment. The number of beneficial outcomes associated with moderate downsizing and moderate expansion in the public sector outweighed the number of adverse outcomes. However, in the private sector the overall effect of moderate organizational change was a poorer work environment.”* [22].

Our findings add to the previous research by suggesting different patterns of reorganizations, rounds of layoffs and subsequent considerations of retirement for different job-function categories. *Strength and limitations.*

It is a major strength of this study that the sample has been drawn among all eligible Danish wage earners ≥ 50 years. Furthermore, lack of responses was accounted for by model-assisted weights, which ensures that estimates are representative. This strengthens the generalizability of the study results [17, 23].

In this study, we have in cross-sectional data examined the association between the perceived quality reorganization and the expectations to retirement age, and the expected retirement age is not necessarily in accordance with the actual retirement age. In general, however, indicators for retirement intentions have been positively associated with subsequent retirement, but with a tendency toward later rather than earlier retirement than anticipated [24]. It is likely that perceptions associated with a recent reorganization may fade in intensity, and the association with retirement intentions accordingly will weaken with time. Furthermore, it is possible that the causation goes the other way around. That is, participants who have already decided to retire, are more likely to judge the quality of the change process to be low. It is therefore highly recommended that future studies will prospectively examine the association between perceived quality of reorganization processes and subsequent register-based retirement age.

Data were collected during the Covid-19 pandemic, and this may have influenced the perceptions of reorganizations as well as expected retirement age. Probably toward a more negative perception of the reorganizations and toward a lower expected retirement age. The period for the data collection may also explain the lower rate of rounds of layoffs reported among the employees working with people compared to the two other job-function categories. Work with people within the health sector as well as the social sector was highly valued during this period.

### Recommendations for Practice

Results from this study suggest that organizational changes may counteract retention of older employees, and stress the importance of attention both to the frequency and to the quality of implementation of organizational changes. There may be good reasons for making reorganizations or rounds of layoffs, but awareness should be given to the not intended effects on older employee’s retirement expectations. If reorganization is inevitable, a high-quality implementation process that ensures transparency and legitimacy of the changes, involvement of relevant parties and a high level of communication about the changes, may increase the likelihood that senior employees choose to stay longer in the company, but it will probably contribute just as much to retaining all other employees in the organization as well. In addition, high-quality implementation process may contribute to increasing the social capital at the workplace.

## Conclusion

Results from this study showed that experiences of organizational change processes of moderate or poor quality were associated with expectations of earlier retirement, while well implemented changes were not. Although the numerical differences were of moderate size, the total expected reduction in work-productive years may be considerable, underscoring the importance of a high-quality implementation process when changes at the organizational level are needed.

## Data Availability

The authors encourage the collaboration and use of the data by other researchers. Researchers who are interested in using the data for scientific purposes should contact Lars L. Andersen, lla@nfa.dk.
